# Breaking the barrier: stigma, psychiatric disorders and life-threatening risks in healthcare: case report

**DOI:** 10.1192/j.eurpsy.2025.1580

**Published:** 2025-08-26

**Authors:** A.-L. Comșa, I. C. Mandras, S. A. Rus

**Affiliations:** 1 Psychiatry, County Emergency Clinical Hospital Cluj-Napoca , Cluj-Napoca, Romania

## Abstract

**Introduction:**

The existing studies found that stigma expands also among healthcare professionals when it comes to psychiatric patients, where a particular group is represented by substance use disorder patients, who are often perceived as manipulative, irresponsible or non-compliant.

**Objectives:**

The aim of this case presentation is to emphasize the importance of stigma reduction especially among healthcare professionals.

**Methods:**

A 26-year-old female patient with medical history of treatment-resistant epilepsy and misuse of cannabis and alcohol was brought into the Psychiatry ward after multiple grand-mal seizures, which occurred after weeks of daily use of alcohol and no adherence to the medical treatment.

**Results:**

Prior to the admission, the patient was directed to the Neurology ward, where the hospitalization was declined due to her psychiatric history and the multi-drug test result, which turned positive for THC.

On the first day after admission, the patient had two seizure episodes, lasting 10 and respectively 30 minutes, after which she was transferred to the ICU department, where she was stabilized. Therefore, she returned to the psychiatric ward, where the patient enters status epilepticus, for which she underwent an neurological examination and received emergency treatment successfully.

The following day, the patient presents another episode of status epilepticus, after which she does not recover her respiratory function spontaneously and suffers cardiac arrest. The resuscitation protocol was initiated, an Emergency Medical team was requested to take over. After 4 minutes of CPR, the patient became pulmonary and hemodynamically stable.

**Conclusions:**

Stigma is one of the factors that can influence the quality of the healthcare services provided by physicians. In the given case, stigma led to a life-threatening scenario, in which the patient was denied to receive adequate neurological treatment due to cannabis and alcohol use disorder. The impact of stigma on healthcare delivery and the barriers to receiving adequate treatment in these cases emphasize the need for training and education for all healthcare professionals.

**Disclosure of Interest:**

None Declared

